# Comparison of Hematologic Toxicity and Bone Marrow Compensatory Response in Head and Neck vs. Cervical Cancer Patients Undergoing Chemoradiotherapy

**DOI:** 10.3389/fonc.2020.01179

**Published:** 2020-07-21

**Authors:** Lucas K. Vitzthum, Elena S. Heide, Helen Park, Casey W. Williamson, Paige Sheridan, Minh-Phuong Huynh-Le, Igor Sirak, Lichun Wei, Rafal Tarnawski, Umesh Mahantshetty, Cammie Nguyen, Jyoti Mayadev, Catheryn M. Yashar, Assuntina G. Sacco, Loren K. Mell

**Affiliations:** ^1^Radiation Medicine and Applied Sciences, University of California, San Diego, La Jolla, CA, United States; ^2^Department of Oncology and Radiotherapy, University Hospital in Hradec Kralove, Hradec Kralove, Czechia; ^3^Department of Radiation Oncology, Xijing Hospital, Xi'an, China; ^4^Maria Sklodowska-Curie Memorial Cancer Center and Institute of Oncology, Gliwice, Poland; ^5^Department of Radiation Oncology, Tata Memorial Hospital, Mumbai, India; ^6^Department of Hematology and Oncology, University of California, San Diego, La Jolla, CA, United States

**Keywords:** hematologic toxicity, cervical cancer, head and neck cancer, FDG PET, hematopoieisis, bone marrow toxicity

## Abstract

**Background:** Hematologic toxicity is a critical problem limiting treatment delivery in cancer patients undergoing concurrent chemoradiotherapy. However, the extent to which anatomic variations in radiation dose limit chemotherapy delivery is poorly understood. A unique natural experiment arises in patients with head and neck and cervical cancer, who frequently undergo identical chemotherapy but receive radiation to different regions of the body. Comparing these cohorts can help elucidate to what extent hematologic toxicity is attributable to marrow radiation as opposed to chemotherapy.

**Methods:** In this longitudinal cohort study, we compared hematologic toxicity and bone marrow compensatory response in 148 patients (90 cervix, 58 head/neck) undergoing chemoradiotherapy with concurrent weekly cisplatin 40 mg/m^2^. We used linear mixed effect models to compare baseline and time-varying peripheral cell counts and hemoglobin levels between cohorts. To assess bone marrow compensatory response, we measured the change in metabolically active bone marrow (ABM) volume on ^18^F-fluorodeoxyglucose positron emission tomography/computed tomography.

**Results:** We observed greater reductions in log-transformed lymphocyte, platelet, and absolute neutrophil counts (ANC) for cervix compared to head/neck cancer patients (fixed effects for time-cohort interaction [95% CI]: lymphocytes, −0.06 [−0.09, −0.031]; platelets,−0.028 [-0.051, −0.0047]; ANC, −0.043 [−0.075, −0.011]). Mean ANC nadirs were also lower for cervical vs. head/neck cancer cohorts (2.20 vs. 2.85 × 10^3^ per μL, *p* < 0.01). Both cohorts exhibited reductions in ABM volume within the radiation field, and increases in ABM volume in out-of-field areas, indicating varying compensatory response to radiation injury.

**Conclusions:** Cervical cancer patients had faster decreases in ANC, lymphocyte, and platelet counts, and lower ANC nadirs, indicating a significant effect of pelvic irradiation on acute peripheral blood cell counts. Both cohorts exhibited a compensatory response with increased out-of-field bone marrow activity.

## Introduction

The hematopoietic system is highly sensitive to injury from both cytotoxic chemotherapy and radiation (1–3). Accordingly, hematologic toxicity is a common complication of chemoradiotherapy that can limit the delivery of prescribed treatment (4). Evolution in radiation technology has allowed substantially greater ability to reduce dose to critical normal tissues, such as bone marrow, offering the possibility to preserve hematopoietic function and permit better treatment tolerance in patients undergoing chemoradiotherapy. Bone marrow dose constraints have been integrated into clinical trials and routine practice for cervical cancer patients (5, 6). However, it is unclear whether greater reductions in bone marrow doses, such as can be achieved with proton therapy (7), would further limit hematopoietic toxicity. It is therefore of clinical and scientific interest to better understand how radiation affects peripheral blood cell counts and bone marrow compensatory response in patients undergoing chemoradiotherapy.

A unique natural experiment arises when comparing cervical cancer and head/neck cancer patients, who often undergo identical chemotherapy but have radically different radiation volumes. This phenomenon provides a rare opportunity to isolate hematologic effects specific to radiation in patients treated with chemotherapy. In this study, we examined differences in hematologic toxicity and bone marrow compensatory response between two cohorts receiving concurrent weekly cisplatin (40 mg/m^2^) and radiation to either the pelvis or head/neck region. We hypothesized that cervical cancer patients, who have a higher volume of irradiated bone marrow, would experience greater acute hematologic toxicity and impaired compensatory response.

## Methods

### Study Design, Population, and Sampling Methods

We conducted a longitudinal cohort study of 90 patients with cervical cancer and 58 patients with head/neck cancer. Eligible patients had newly diagnosed, previously untreated, biopsy-proven head/neck or cervical carcinoma, undergoing concurrent chemoradiotherapy with intent to deliver five or more cycles of weekly cisplatin between April 2011 and January 2018. Patients receiving additional concurrent systemic or (neo)adjuvant therapy (i.e., non-platinum cytotoxic chemotherapy, immunotherapy, targeted therapy, etc.), were non-adherent to treatment, were postoperative, or had previous radiation at the site within the last 5 years were excluded. This study was a retrospective analysis of de-identified patient data. The University of California San Diego Human Research Protections Program approved this study and determined that it was of minimal risk and did not meet the criteria for Human Subjects Research.

### Treatment

Chemotherapy consisted of five to six cycles (cervix) or seven cycles (head/neck) of cisplatin 40 mg/m^2^ delivered concurrently with external beam radiation therapy. Cisplatin was held under the following conditions: white blood cell count (WBC) <2.0 × 10^9^/L, absolute neutrophil count (ANC) × 1.0 × 10^9^/L, hemoglobin <7.5 g/dL, platelet count <50 × 109/L, or creatinine clearance <50 mL/min. Head/neck patients in this cohort were selected for weekly over triweekly cisplatin based on the preference of the treating medical oncologist. Granulocyte colony stimulating factor (G-CSF) and/or transfusions were given at the discretion of the treating medical oncologist.

Radiotherapy for cervical cancer consisted of volumetric modulated arc therapy (VMAT) with 45.0–47.6 Gy to the pelvis and 53.2–59.4 Gy to grossly involved lymph nodes over 5–6 weeks, followed by intracavitary brachytherapy, 24.0–30.0 Gy to the cervical tumor. Brachytherapy doses were not given during the initial 5 weeks of radiation and therefore did not contribute to dose during the study's observation period. Radiotherapy for head/neck cancer consisted of VMAT with 54.0–63.0 Gy to the neck and 70.0 Gy to gross disease over seven weeks. Bone marrow dose constraints were not used for head/neck cancer. The constraints for cervical cancer were 90% of the volume receiving <10 Gy (V_10Gy_ < 90%) and V_20Gy_ < 75%.

### Hematologic Toxicity and Compensatory Response

We collected weekly complete blood counts with differentials for all patients beginning immediately prior to the initiation of chemoradiotherapy through the end of treatment. To standardize and control for the number of cisplatin cycles given, we analyzed ANC, lymphocyte, hemoglobin, and platelet counts up to 5 weeks following initiation of treatment.

To assess compensatory response, we calculated the change in metabolically active bone marrow (ABM) volume in a subset of 33 patients (19 cervical, 14 head/neck) who underwent pre- and post-treatment imaging with ^18^F-fluorodeoxyglucose positron emission tomography / computed tomography (FDG-PET/CT). Whole body FDG-PET/CT scans extended from the top of the skull through mid-femur. Pre-treatment FDG-PET/CT scans were acquired within 3 months prior to the initiation of chemoradiation, whereas post-treatment scans were acquired 3–6 months following the conclusion of treatment. The median from the end of treatment to the post-treatment FDG-PET/CT scan for head/neck and cervical cancer patients was 15.7 vs. 14.7 weeks (*p* = 0.29), respectively.

We used the MIM platform (MIM Software, Inc., Cleveland, OH) to delineate external contours of the following regions on each patient's baseline and follow-up FDG-PET/CT: clavicle/sternum, scapula/proximal humerus, ribs, cervical spine, thoracic spine, L1 through L4, L5/sacrum, ileum, and ischium/pubes/proximal femora. The cumulation of all these bone structures was defined as the total bone. Subsequently, the mean body weight-corrected standardized uptake value (SUV) was quantified within total bone, and ABM was identified as sub-region with an SUV greater than the individual patient's mean SUV (for each scan) within the total bone, as previously described (8, 9). The total bone volume (TBV) and ABM volume (ABMV) were calculated.

Bone and ABM dose calculations were performed using deformable registration to align the baseline FDG-PET/CT to the treatment planning CT scan and corresponding radiation plan.

### Statistical Analysis

The primary endpoint was change in ANC from baseline to week 5 of treatment. Secondary endpoints were changes in lymphocyte, hemoglobin, and platelet counts, and change in ABM volume both within and outside of the radiation field. Baseline covariates were compared between cohorts using chi-square tests for categorical variables and unpaired *t*-tests for continuous variables. Nadir cell counts were compared between cohorts using unpaired two-tailed *t*-tests. The number of patients requiring blood transfusion, a G-CSF or erythropoietin were compared between groups using Fisher's exact test.

Linear mixed-effects models were used to estimate longitudinal changes in (log-transformed) blood counts, with a subject-specific random intercept and random slope. We allowed the rate of change over time to differ by sex and cancer type, by including interactions between these terms and follow-up time. Cell count values were log-transformed to approximate a normal distribution. Fixed effects included in the models were sex, time (weeks), cohort (cervical vs. head/neck), and interaction between time and cancer type. Restricted maximum likelihood was used to estimate fixed effect parameters. Backward stepwise techniques were used to select covariates including gender, age, cancer type, time and interactions between gender and time and cancer type and time (*p* < 0.20) (10). In an exploratory analysis, we also evaluated whether low (I-II) vs. high (III-IV) FIGO stage was associated with longitudinal changes in blood counts for cervical cancer patients.

## Results

The cervix cohort included 77 patients (85.6%) with stage I-II and 13 (14.4%) with stage III-IVA disease (International Federation of Gynecology and Obstetrics 2009 edition). The head/neck cohort comprised five patients (8.6%) with stage I-II and 53 patients (91.4%) with stage III-IVB disease (American Joint Committee on Cancer 7th edition). The head/neck cohort comprised 18 females (31%) and 40 males (69%) with carcinoma of the nasopharynx (3.4%), nasal cavity (3.4%), salivary gland (3.4%), hypopharynx (6.9%), larynx (6.9%), oral cavity (17.2%), or oropharynx (58.6%).

Compared to the head/neck cohort, the cervix cohort was younger (51.9 vs. 63.0 years, *p* < 0.01) and had a significantly lower percentage of patients that were non-Hispanic white (57.8 vs. 84.5%, *p* < 0.01) (Table 1). There was a similar mean ABM dose between groups (17.8 vs. 15.4 Gy, *p* = 0.22) but a significantly higher volume of ABM receiving at least 5 Gy (V_5_: 516.8 vs. 301.5 cm^3^, *p* < 0.01) and 10 Gy (V_10_: 442.5 vs. 255.7 cm^3^, *p* < 0.01) in cervical cancer patients.

**Table 1:** Sample characteristics.

**Characteristic**	**Cervical cancer**	**Head/Neck cancer**	***p***
Number	90	58	
Age, years	51.9 (11.7)	63.0 (9.0)	<0.01
Body mass index, kg/m^2^	27.4 (5.5)	25.9 (5.6)	0.14
**Sex**, ***n*** **(%)**			
Male	0 (0%)	40 (69.0%)	<0.01
Female	90 (100%)	18 (31.0%)	
**Race**, ***n*** **(%)**			
White	52 (57.8%)	49 (84.5%)	<0.01
Black	3 (3.3%)	1 (1.7%)	
Hispanic/Latino(a)	13 (14.4%)	6 (10.3%)	
Asian	19 (21.1%)	2 (3.4%)	
Other	3 (3.3%)	0 (0%)	
**Cycles of cisplatin held up to week 5**, ***n*** **(%)**			
0	81 (90.0%)	56 (96.6%)	0.13
1	7 (7.8%)	2 (3.4%)	
2	1 (1.1%)	0 (0.0%)	
3	0 (0.0%)	0 (0.0%)	
4	1 (1.1%)	0 (0.0%)	
Active bone marrow dose, Gy	17.8 (4.9)	15.4 (4.6)	0.22
Active bone marrow V_5_, cc	516.8 (78.9)	301.5 (97.1)	<0.01
Active bone marrow V_10_, cc	442.5 (65.6)	255.7 (90.2)	<0.01

*Values are means (standard deviation), unless otherwise indicated. V_5_, volume receiving dose of 5 Gy or more; V_10_, volume receiving dose of 10 Gy or more*.

**Figure 1:**
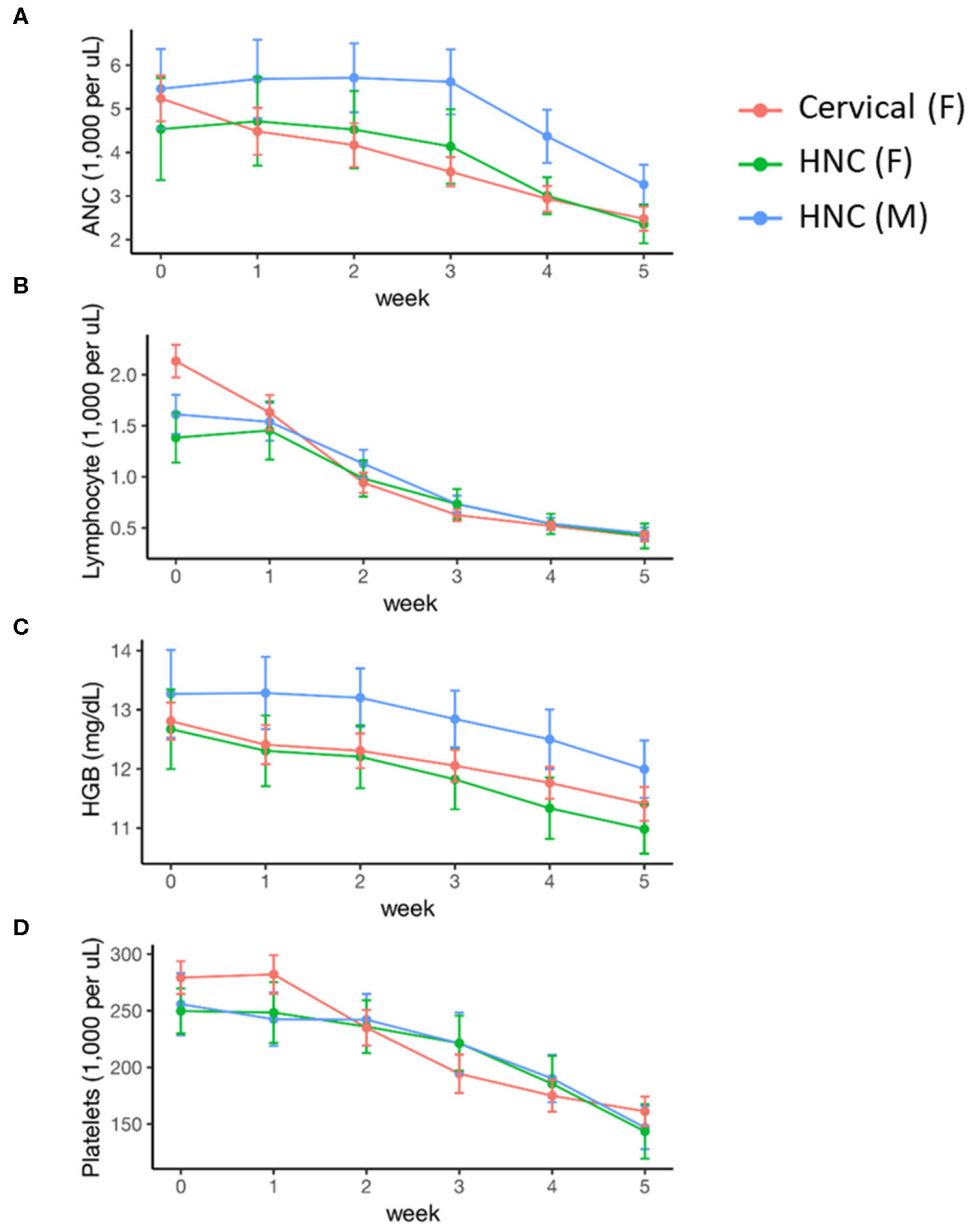
Acute hematologic response during definitive chemoradiation, by disease and sex. **(A)** Absolute neutrophil counts (ANC); **(B)** lymphocyte counts; **(C)** hemoglobin (HGB) levels; **(D)** platelet counts. HNC, Head and Neck Cancer; M, Male; F, Female.

Female sex was associated with lower baseline ANC and hemoglobin levels, but there was no significant difference in these values by disease cohort (Table 2). Cervical cancer was associated with significantly higher baseline platelet levels (0.094 [0.008, 0.18]). Peripheral cell counts significantly declined over the course of treatment in each cohort (Figure 1; Table 2). BMI did not correlate with baseline cell count values or change over time on multivariable analysis. Compared to the head/neck cohort, the cervix cohort had a lower mean ANC nadir (2.20 vs. 2.85 × 10^3^ per μL, *p* < 0.01), and had greater mean reductions in ANC, lymphocyte, and platelet counts with treatment (Figure 1; Table 2). Nadir values were not significantly different between cohorts for lymphocyte, hemoglobin, or platelet counts, and there was no significant interaction between time and disease cohort for hemoglobin levels. We observed no significant interactions between gender and time for any of the peripheral cell counts. Patient age (coded as continuous or age ≥65) was not associated with peripheral cell count values at baseline or with changes over time.

**Table 2:** Linear mixed effect model for longitudinal changes in cell counts.

**Fixed effect**	**ANC**	**Lymphocytes**	**Hemoglobin**	**Platelets**
Intercept	**1.80 (1.70, 1.90)**	**0.55 (0.45, 0.64)**	**2.60 (2.60, 2.60)**	**5.60 (5.50, 5.70)**
Time (weeks)	**−0.10 (−0.13**, **−0.078)**	**−0.29 (−0.31**, **−0.26)**	**−0.021 (−0.027**, **−0.014)**	**−0.11 (-0.13,−0.085)**
Cervical Cancer (Ref: Head/Neck)	0.045 (–0.081, 0.17)	**0.24 (0.12, 0.36)**	0.031 (–0.0014, 0.063)	**0.094 (0.008, 0.18)**
Female (Ref: Male)	**−0.24 (-0.36**, **−0.13)**	**−0.14 (−0.25**, **−0.033)**	**−0.078 (−0.11**, **−0.048)**	–0.032(–0.11, 0.045)
Time x Cervical Cancer	**−0.043(-0.075**, **−0.011)**	**−0.060(-0.090**, **−0.031)**	–0.001 (-0.009, 0.007)	**−0.028(-0.051**, **−0.005)**

*Effects are on log-transformed values of the peripheral cell counts. Bold face indicates statistical significance (p < 0.05). ANC, absolute neutrophil count*.

In the cervix cohort, nine patients (10.0%) had one or more cycles of chemotherapy held during the 5 weeks of treatment, compared to two patients (3.4%; *p* = 0.13) in the head/neck cohort. Reasons for holding cisplatin were documented in three cervix patients, and included nephrotoxicity, neutropenia, and patient refusal. Reasons for holding cisplatin in the two head/neck patients were hypovolemia and severe nausea requiring feeding tube placement. None of the head/neck cancer patients were treated with G-CSF compared to five (5.6%) cervical cancer patients (*p* = 0.16). No patients from either cohort were treated with erythropoietin. One (1.8%) head/neck cancer patient underwent packed red blood cell (PRBC) transfusion compared to eight (8.9%) cervical cancer patients (*p* = 0.09). None of the head/neck cancer patients required platelet transfusion, compared to one (1.1%) cervical cancer patient (*p* = 1). On exploratory analysis, there was no correlation between FIGO stage and cell counts at baseline or over time (results not shown).

**Figure 2:**
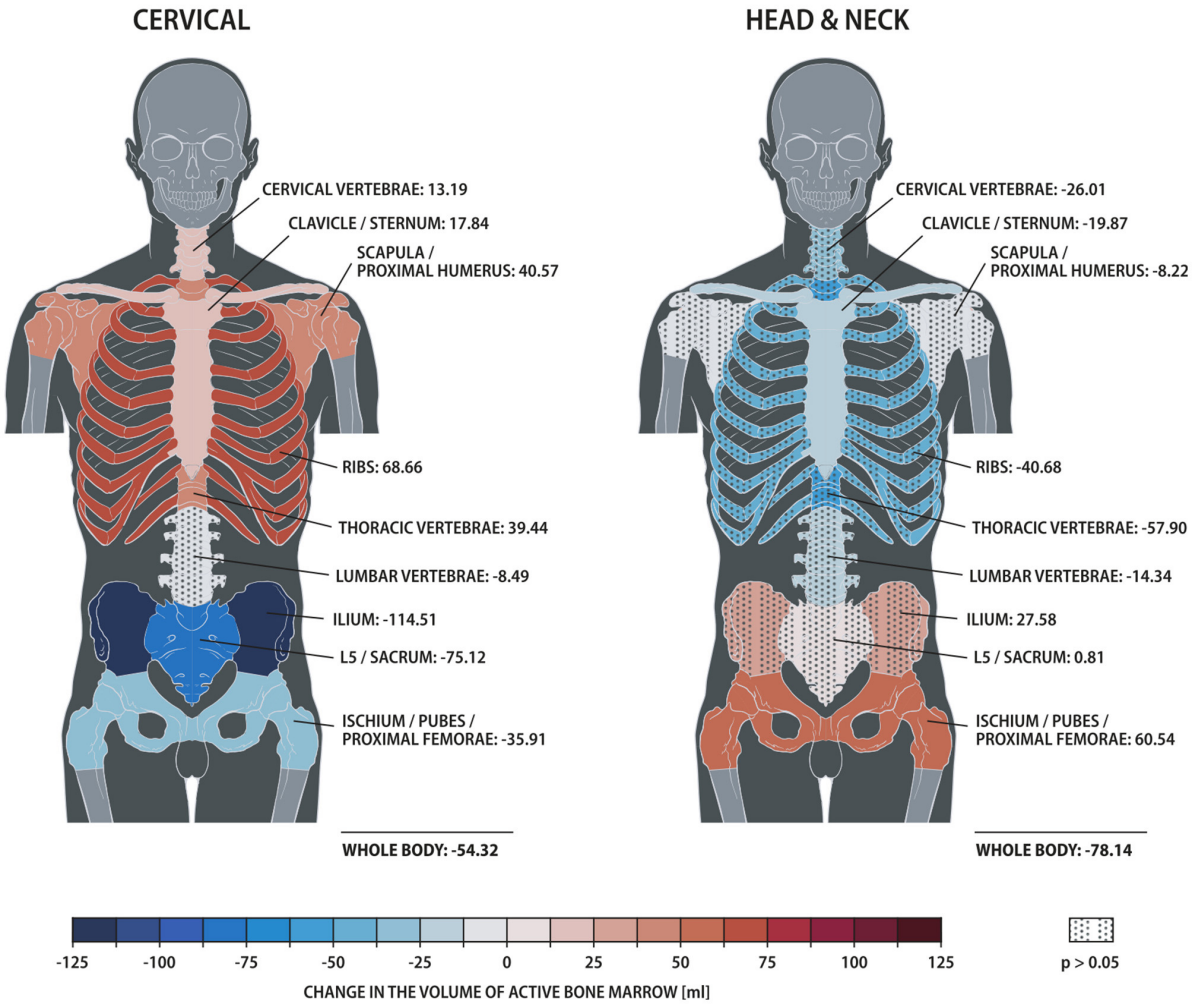
Heat map comparing changes in active bone marrow volume within skeletal sub-structures, measured by ^18^F-FDG PET/CT, for patients treated for cervical vs. head/neck cancer. Solid and dotted fill represent *p* < 0.05 and ≥0.05, respectively.

The distribution of ABM changed significantly with treatment for both cervical and head/neck cancer patients (Figure 2, Table 3). Cervical cancer patients had a significant decrease in mean ABM volume in the pelvis and a significant increase in mean ABM volume in the cervical vertebrae, thoracic vertebrae, clavicle, scapula and proximal humeri and ribs. Among head/neck cancer patients, there was a significant decrease in mean ABM volume in the clavicle and sternum and a significant increase in mean ABM volume in the pelvis.

**Table 3:** Change in active bone marrow volume (compensatory response), by cancer type.

	**Total bone marrow volume (cm**^****3****^**)**		**ABM pre-treatment volume (cm**^****3****^**)**		**ABM post-treatment volume (cm**^****3****^**)**		**Change in ABM volume (cm**^****3****^**)**	
**Region**	**Cervical**	**Head/Neck**	**Cervical**	**Head/Neck**	**Cervical**	**Head/Neck**	**Cervical**	**Head/Neck**
Clavicle	128.9 (22.4)	209.5 (38.6)	43.9 (16.3)	78.4 (35.7)	61.7 (20.0)	58.5 (29.4)	**17.8 [*****p*** **<** **0.01]**	**−19.9 [*****p*** **=** **0.02]**
Pelvis	1230.2 (184.6)	1743.2 (363.8)	504.7 (107.5)	608.9 (224.7)	279.1 (127.6)	697.9 (194.3)	**−225.5 [*****p*** **<** **0.01]**	88.9 [*p* = 0.05]
Ribs	636.3 (15.3)	1062.8 (277.3)	135.9 (61.2)	342.7 (111.4)	204.5 (68.4)	302 (75.2)	**68.7 [*****p*** **<** **0.01]**	–40.7 [*p* = 0.11]
Scapula, Proximal Humerus	377.9 (62.0)	645.4 (144.6)	77.8 (46.8)	127.7 (74.8)	118.4 (48.5)	119.5 (91.6)	**40.6 [*****p*** **<** **0.01]**	–8.2 [*p* = 0.65]
Cervical Spine	127.3 (25.0)	173.5 (45.8)	111.2 (24.3)	148.8 (39.8)	124.4 (21.4)	122.7 (41.2)	**13.2 [*****p*** **=** **0.01]**	–26.1 [*p* = 0.20]
Thoracic Spine	378.2 (58.7)	555.2 (125.7)	273.8 (45.8)	475.6 (102.4)	313.2 (46.2)	417.7 (126.1)	**39.4 [*****p*** **<** **0.01]**	−57.9 [*p* = 0.08]
Lumbar Spine	282.2 (40.7)	380.6 (94.4)	179.9 (39.4)	312.1 (87.2)	171.4 (35.8)	297.7 (109.8)	–8.5 [*p* = 0.30]	–14.3 [*p* = 0.60]
Whole Body	3155.5 (518.1)	4757.7 (1022.1)	1327.1 (219.2)	2094.1 (512.6)	1272.8 (201.3)	2016.0 (450.2)	**−54.3 [*****p*** **=** **0.03]**	–78.1 [*p* = 0.12]

*ABM, active bone marrow. Bold values indicate p-value < 0.05*.

## Discussion

Ionizing radiation results in hematopoietic toxicity through impairment of bone marrow production and redistribution and apoptosis of mature circulating cells (11). Acute hematologic toxicity is a common side effect in patients undergoing chemoradiotherapy that can result in morbidity and poor treatment tolerance (12–16). Previous studies have found that hematologic toxicity varies as a function of bone marrow radiation dose, volume, and location (5, 10, 13, 17–23). These studies have led to the development of bone marrow radiation dose constraints that have been incorporated into clinical trials and routine practice for cervical cancer patients (5, 6). It is not known whether additional reduction in marrow radiation, on par with levels in head/neck cancer treatment, would further reduce toxicity or whether benefits would outweigh risks associated with potentially increased dose to other organs. Prior dosimetric studies have shown that current bone marrow constraints are feasible, however, greater optimization for bone marrow avoidance could result in increased dose to adjacent organs at risk (24, 25).

Radiation is also associated with imaging changes on both MRI and FDG-PET/CT (21, 26). However, studying the clinical effects of radiation dose on hematopoiesis is challenging, particularly in the context of concurrent chemotherapy delivered at radiosensitizing doses, due to limitations on the degree of dose variation to bone marrow that is feasible or ethical.

Here we took advantage of a natural experiment arising from standard clinical practice patterns to analyze patients treated with identical chemotherapy who received concurrent radiation targeted to radically different parts of the anatomy. As such, we attempted to isolate the effect of varying radiation volume on bone marrow dose, acute hematologic toxicity, and hematopoietic compensatory response, while controlling for chemotherapy dosing. We found that, compared to patients with head/neck cancer, patients with cervical cancer had lower ANC nadirs and a more pronounced rate of decline in ANC, lymphocyte, and platelet counts over the first 5 weeks of treatment, along with higher volumes of marrow irradiated, indicating that the radiation volume was a contributing factor affecting peripheral cell counts. However, the difference between rate of decline was modest and head/neck cancer patients also had significant reductions in each of the cell types we studied, indicating that systemic therapy is still a predominant cause of reduced peripheral counts in the acute setting. These findings suggest that further efforts to spare BM beyond currently employed dose constraints and techniques with IMRT may have a limited impact on hematopoietic toxicity. Of note, we observed no statistically significant differences in the number of patients requiring treatment with G-CSF, PRBCs, or platelet transfusions.

Both cervical and head/neck cancer patients had greater decreases in ABM volume in irradiated bone sub-regions, with corresponding increased metabolic activity in unirradiated sites. However, by virtue of having less marrow irradiated, head/neck cancer patients are likely better able to compensate for hematopoietic injury in association with chemoradiotherapy compared to patients with cervical cancer. Prior studies have found that proliferative activity of out-of-field myeloid stem cells increases in response to radiation (27–29). Varying patterns of compensatory response reflect different capacity to respond to bone marrow injury, which in turn has been correlated with both acute hematologic toxicity and intensity of chemotherapy (9). Note, however, we were only able to quantify the subacute compensatory response (measured three to 6 months post-treatment) in this study, and future studies with longitudinal FDG-PET/CT to monitor the acute compensatory response (e.g., at week five of chemoradiation) would be valuable. There is also evidence that even low doses of radiation to active bone marrow can lead to reductions in WBC counts that persist for months after treatment (30). By limiting our analysis of blood counts to the first 5 weeks of treatment (due to differing lengths of chemoradiation between disease sites) we are not able to assess longer term trends in cell counts and sub-acute hematologic toxicity.

Serial FDG-PET/CT is a useful method to study both hematopoietic activity and compensation for marrow injury, as regions of higher metabolic activity within the marrow are correlated with higher cellularity and proliferative activity (31–33). Previous studies also support the hypothesis that radiation techniques designed to reduce dose specifically to metabolically active bone marrow, as measured by FDG-PET/CT, can reduce acute hematologic toxicity and permit better chemotherapy tolerance, with ongoing clinical trials continuing to investigate this therapeutic strategy (5, 20, 22). Preserving hematopoietic progenitor cells may also be increasingly important as radiation therapy is being used in conjunction with anticancer immunotherapy agents (7, 34).

There are several limitations to this study. As this was a retrospective analysis, unmeasured population differences between cohorts, or males and females, could explain our findings, independent of bone marrow irradiation. It was challenging to elucidate the effect of gender vs. RT distribution when comparing a cohort of cervical vs. predominately male head/neck cancer patients. However, we did control for measurable covariates with multivariable analysis. Furthermore, randomizing patients to widely different radiation volumes would be unethical in the population we studied. Additional unmeasured variables, including patient comorbidity and smoking status, can effect hematopoietic response and may be a source of confounding. Currently, no satisfactory methods exist to measure and control for radiation effects on circulating hematopoietic progenitor cells, which could also contribute to the observed reductions in peripheral cell counts. This cohort of cervical and head/neck cancer patients is heterogeneous with varying stages, subsites and radiation plans. Variation in radiation dose distribution within the head/neck or cervical cancer groups may limit the ability to identify differences in hematologic toxicity or compensatory response between groups.

Serial FDG-PET/CT images were acquired through the natural course of care and were thus only available for a subset of our sample; future research should attempt to correlate changes in peripheral cell counts with the acute compensatory response. It would also be of academic interest to evaluate bone marrow changes in the absence of systemic therapy, although prior data indicates that pelvic RT alone rarely results in hematologic toxicity (19, 35). Comparison of FDG-PET between the groups may also be confounded by differences in total radiation and chemotherapy exposure prior to the post-treatment scan. Interestingly, only one cervical cancer patient was identified as having chemotherapy held for cytopenia. Additional research is needed to determine if the differences in peripheral cell counts from varying radiation dose distribution described in this study result in differences in patient-reported outcomes (36).

In conclusion, we found that cervical cancer patients had lower ANC nadirs and faster decreases in ANC, lymphocyte, and platelet counts over the first 5 weeks of treatment, compared to head/neck cancer patients treated with identical chemotherapy. Both cohorts exhibited different patterns of subacute compensatory response in the bone marrow, and the higher volume of active marrow irradiated in cervical cancer patients appears to be a significant independent contributor to a faster depletion of circulating neutrophil, lymphocyte and platelet cell types. Cervical cancer patients also had a lower average ANC. Further studies are needed, however, to characterize the relationship between varying radiation dose distributions and hematologic response in patients undergoing concurrent chemotherapy.

## Data Availability Statement

The raw data supporting the conclusions of this article will be made available by the authors, without undue reservation pending institutional approval.

## Ethics Statement

This study was a retrospective analysis of de-identified patient data. The University of California San Diego Human Research protections program approved this study and determined that it was of minimal risk and did not meet the criteria for Human Subjects Research.

## Author Contributions

LM contributed to the study design, statistical analysis, and manuscript writing and editing. EH contributed to the study design, data collection, statistical analysis, manuscript writing, and editing. HP contributed to data collection and statistical analysis. CW contributed to data collection, statistical analysis, and manuscript writing and editing. PS contributed to statistical analysis and manuscript editing. M-PH-L contributed to statistical analysis. IS, LW, RT, JM, CY, and AS contributed to manuscript writing and editing. CN contributed to data collection and analysis. LM contributed to study design, statistical analysis, and manuscript writing and editing. All authors contributed to the article and approved the submitted version.

## Conflict of Interest

The authors declare that the research was conducted in the absence of any commercial or financial relationships that could be construed as a potential conflict of interest.
